# Alcohol Industry CSR Organisations: What Can Their Twitter Activity Tell Us about Their Independence and Their Priorities? A Comparative Analysis

**DOI:** 10.3390/ijerph16050892

**Published:** 2019-03-12

**Authors:** Nason Maani Hessari, May CI van Schalkwyk, Sian Thomas, Mark Petticrew

**Affiliations:** 1 Department of Health Services Research and Policy, London School of Hygiene and Tropical Medicine; London WC1H 9SH, UK; nason.maani-hessari@lshtm.ac.uk; 2 Department of Primary Care and Public Health, Imperial College London W6 6RP, UK; m.van-schalkwyk@imperial.ac.uk; 3 Department of Public Health, Environments and Society, London School of Hygiene and Tropical Medicine, London WC1H 9SH, UK; sian.thomas@btinternet.com

**Keywords:** alcohol industry, public health, cancer, social media, thematic analysis

## Abstract

There are concerns about the accuracy of the health information provided by alcohol industry (AI)-funded organisations and about their independence. We conducted a content analysis of the health information disseminated by AI-funded organisations through Twitter, compared with non-AI-funded charities, to assess whether their messages align with industry and/or public health objectives. We compared all tweets from 2016 from Drinkaware (UK); Drinkaware.ie (Ireland); and DrinkWise (Australia), to non-AI-funded charities Alcohol Concern (UK), Alcohol Action Ireland, and FARE (Australia). Industry-funded bodies were significantly less likely to tweet about alcohol marketing, advertising and sponsorship; alcohol pricing; and physical health harms, including cancers, heart disease and pregnancy. They were significantly more likely to tweet about behavioural aspects of drinking and less likely to mention cancer risk; particularly breast cancer. These findings are consistent with previous evidence that the purpose of such bodies is the protection of the alcohol market, and of the alcohol industry’s reputation. Their messaging strongly aligns with AI corporate social responsibility goals. The focus away from health harms, particularly cancer, is also consistent with previous evidence. The evidence does not support claims by these alcohol-industry-funded bodies about their independence from industry.

## 1. Introduction

As part of their corporate social responsibility (CSR) activities, alcohol companies fund a range of social aspects/public relations organisations (SAPROs). The stated aims of such organisations are to educate the public and promote “responsible drinking” [1,2], but a growing body of evidence indicates they exist primarily to improve public relations, distract from evidence-based policies and resultant legislation that might be harmful to profits, emphasize personal over corporate responsibility, and mislead the public on health risks [3,4,5,6,7,8]. This is consistent with the global alcohol producers’ commitments, which have been a focus of alcohol industry (AI) corporate social responsibility messaging, and are cited by industry as evidence of a commitment to meaningful social contribution, yet avoid population-level measures listed in the WHO’s global alcohol strategy (see Table 1), which are most likely to reduce overall alcohol consumption [9,10] and, therefore, negatively affect profits [11,12]. 

Even in instances of apparent overlap in priorities between the WHO and global producer commitments, evidence is emerging in areas such as strengthening marketing regulation [15,16], reducing drink driving [17], or providing information to the public [18,19] that the majority of industry initiatives are not only inconsistent with available evidence of effectiveness [5], but often occur alongside industry opposition to evidence-based policies [20,21]. In spite of this conflict, in many countries SAPROs are the main source of information on alcohol and health for consumers [3].

In the UK, Ireland and Australia, the main AI-funded SAPROs serving as providers of health information on alcohol are Drinkaware, Drinkaware.ie and DrinkWise, respectively. 

Drinkaware (UK) first appeared in 2004 as an alcohol information website developed by the Portman Group. The Portman Group is an alcohol-producer-funded organization, and at the time, the UK government’s strategy for reducing alcohol-related harms had assigned them responsibility for the provision of information on alcohol to the public [22]. The Drinkaware Trust was then established as a separate charity in 2007, following a 2006 memorandum of understanding between the alcohol industry and a number of government agencies [4], and remains funded primarily by alcohol producers and sellers [23]. Its stated purpose is to “work in partnership with others to help reduce alcohol-related harm by helping people make better choices about their drinking” [23]. Drinkaware and other SAPROs were recently found to be misleading the public on alcohol and cancer risk, including presenting misleading information about the independent effects of alcohol consumption on cancer risk, using similar framings to those developed by the tobacco industry to obscure the evidence on smoking and lung cancer, in some cases not mentioning cancer in general, and breast cancer specifically [19]. Such organisations also place considerable emphasis on potential confounders in the alcohol-cancer relationship, minimising the role of alcohol by pointing to a wide range of other non-modifiable risk factors for cancer [19]. Emphasis is also placed on how knowledge of mechanisms is incomplete, apparently as a way of disputing the relationship. For example, “Scientists have not identified any single mechanism that explains exactly why alcohol increases the risk of developing cancer” (Drinkaware) [19] and “Although alcohol is a well-established risk factor for breast cancer, the mechanism by which alcohol consumption may cause breast cancer is not fully known. The relationship between alcohol consumption and breast cancer is undergoing vigorous research” (SAB Miller) [19]. This can be compared with tobacco industry arguments about smoking and lung cancer—e.g., “Science has still to ascertain precise biological mechanisms whereby prolonged exposure to constituents of tobacco smoke causes these diseases” [24]. More recently, a controversial partnership between Drinkaware and Public Health England (PHE) attracted widespread criticism from the public health community [25].

Drinkaware.ie is a national not-for-profit organization in Ireland, which, similar to Drinkaware (UK), is funded primarily by alcohol industry contributions, and has as a stated mission … “to work with others to fundamentally and permanently change attitudes and behaviours so that drinking to excess and drinking underage become unacceptable.” [26] Drinkaware.ie was established as the consumer-facing brand of the Mature Enjoyment of Alcohol in Society (MEAS), itself established in 2002 as an independent not-for-profit by representatives of the Irish alcohol industry, with responsibility for developing a code of practice for alcohol marketing and for funding educational campaigns directed at the public and at alcohol retailers [27].

DrinkWise (Australia) was established in 2005 by the alcohol industry as a not-for-profit organization, with the stated aim … “to help bring about a healthier and safer drinking culture in Australia.” [28]. Unlike Drinkaware (UK) and Drinkaware.ie, DrinkWise initially was also in receipt of government funding for the first four years of its existence [3]. The independence, evidence-base for activity and transparency of DrinkWise have been challenged by public health researchers who have suggested that Drinkwise’s focus is on individually-oriented research and on social marketing and education campaigns, which the evidence shows are of limited effectiveness [29]. Moreover, recent research on Drinkwise points to confusion amongst the public about its industry funding [30], and presents evidence that current campaigns may be reinforcing social norms relating to heavy drinking among young people [31]. 

All three organisations are public sources of alcohol-related information, and the extent to which their content matches WHO-recommended initiatives or alcohol industry producer initiatives, particularly in terms of responding to seasonal or one-off issues at the national level, is not well understood. Social media is an important communication platform for such charities to interact with followers, advocate, lobby, build interest and launch campaigns [32,33,34,35]. It has also been identified as a potentially rich data source for health research, with a recent systematic review identifying 137 research articles using Twitter, 108 of which involved the analysis of tweets, primarily in the form of content analysis [33]. The strengths of analysing tweets in particular are ease of access and the real-time nature of content generation and distribution. 

We therefore sought to compare the Twitter output of these industry-funded organisations to a leading national alcohol charity in each country (Alcohol Concern, Alcohol Action Ireland and FARE), to assess the extent to which messages align with industry and/or public health objectives. The inclusion of UK, Ireland, and Australia kept the task manageable, while allowing for a direct comparison between major industry-funded, and non-industry funded sources.

## 2. Materials and Methods 

We downloaded and coded all tweets from January-December 2016 from the AI-funded sources Drinkaware (UK, *n* = 835 tweets); Drinkaware.ie (Ireland, *n* = 232); and DrinkWise (Australia, *n* = 89). The sources of non-AI-funded tweets were Alcohol Concern (UK, *n* = 730), Alcohol Action Ireland (*n* = 475), and FARE (Foundation for Alcohol Research and Education, Australia, *n* = 444). After an initial pilot coding exercise in which three coders blind-coded 50 tweets randomly-selected using the random number generator in Excel, coding was conducted independently by two coders, consulting with others where a code was unclear. Where older tweets were unavailable on Twitter we downloaded them from an online Twitter archive. We were primarily interested in determining which health topics AI-funded organisations tweeted about, compared to independent non-industry organisations, because of previous findings that AI-funded bodies present misleading health information, particularly information on cancer, to the public [8,19]. A coding frame was developed by iteratively coding all tweets by topic based on text content, and other characteristics of the tweets, particularly the content of the images in the tweets (whether the tweet contained images of women, young women, children, young adults, people drinking or alcoholic beverages). The coding was recorded in Microsoft Excel (Excel 2016, Microsoft, Redmond, Washington, USA). This form of content analysis is common in health research, and has also been used frequently in the analysis of corporate communications, for example as part of policy debates [36,37,38]. The analysis and reporting adheres to guidance on the collection, analysis and presentation of Twitter data [39].

We developed a series of hypotheses *a priori* based on previous evidence of AI strategies and campaigns. If AI-funded bodies are independent of the alcohol industry (as is argued by Drinkaware, see for example [40]), then no clear pattern should be observed, and their tweets should not reflect alcohol industry positions. On the other hand if, as has been noted previously [41], these bodies exist primarily to reflect and defend industry positions, then their Twitter activity should reflect industry arguments, concerns and topics, and should be significantly different from those of non-industry affiliated charities.

Our prior hypotheses were: 

H1: The topics covered by AI-funded bodies would be similar to the Global Alcohol Producers Commitments [42]. These are: reducing under-age drinking; strengthening and expanding marketing codes of practice; providing consumer information and responsible product innovation; reducing drinking and driving; and enlisting the support of retailers to reduce harmful drinking.

H2: That tweets by AI-funded bodies would have a focus on behavioural aspects of drinking and drink-related harms (i.e., visible antisocial behaviour, rather than chronic health harms), as has been found previously [18];

H3: That AI-funded bodies would be less likely to tweet warning consumers about pregnancy and related issues, and about cancers [8,19]. Since the number of tweets about cancer is low overall, we also included all cancer related tweets from 2017, as this would allow the frequency of tweeting about specific types of cancer to be analysed;

H4: That Twitter communications by AI-funded bodies would be primarily addressed to young women (because visible public drinking and alcohol-related anti-social behaviour in young women is a PR risk to the industry [18]);

H5: That they would emphasis non-regulatory and self-regulatory initiatives [15];

H6: That tweets by AI-funded bodies would show evidence of normalisation of new drinking occasions (e.g., work drinks days), because of evidence that industry CSR ‘responsible drinking’ campaigns can have the dual effect of promoting drinking [2,43], and

H7: Related to H6, that Twitter activity of AI-funded bodies would be more likely to include alcohol, and drinking-related images.

H8: For the analyses of images in the tweets, based on previous research and knowledge of AI priorities we hypothesised that images in tweets by AI-funded bodies would be more likely to include women, and young women in particular; children; images of alcoholic drinks, and images of people drinking.

The data were analysed in SPSS (SPSS Statistics for Windows, Version 24.0. Armonk, NY, USA: IBM Corp). 

We used Z tests to analyse the most common 20 topics, comparing industry-related to non-industry related bodies. Two-tailed *p*-values and 95% confidence intervals for the difference in proportions are presented.

## 3. Results

### 3.1. Comparison of Themes

After coding, the tweets were grouped into forty-five topics across all sources (i.e., both industry-funded and non-industry-funded), plus an ‘other’ category (i.e., where individual tweets could not be coded to any of the 45 topics). Similar tweets were coded into the same category, then the categories ordered by overall frequency. The twenty most common topics tweeted about are broken down by organisation in Table 2, and listed in order of overall frequency of appearance (for the full list of 45 topics see Supplementary Table S1). 

Table 3 compares the topics covered by AI-funded organisations as a group, with non-AI-funded organisations (charities and independent not-for-profit bodies) in terms of these top twenty topics. It shows that in every health-related topic area except two (mental health and calories/obesity), AI-funded organisations were significantly less likely to tweet about that topic.

For example, AI-funded bodies were significantly less likely to tweet about alcohol marketing, advertising and sponsorship; issues related to alcohol pricing, including MUP; physical health harms, including cancers, heart disease, dementia and diabetes; and fertility and pregnancy. They were less likely to tweet about anger/aggression as a consequence of drinking too much; and about the impact of alcohol on emergency services. 

Alcohol industry-funded bodies were significantly more likely to tweet about drinking too much, cutting down, children and underage drinking, teens/parents, staying safe while drinking, alcohol units and guidelines, calories/obesity, and alcohol-free or low alcohol drinks. They are also more likely to tweet about drink driving.

Consistent with the initial hypotheses, they are significantly more likely to tweet about issues related to the Global Alcohol Producers “Producers Commitments to Reduce Harmful Drinking” (especially underage drinking, drinking and driving), behavioural aspects of drinking (anger/aggression; drink driving; “cutting down/cutting back”); and informational aspects—e.g., knowing about guidelines and units. 

### 3.2. Cancer-Related Tweets

One hypothesis was that industry-funded bodies would tweet less about specific types of cancer, given that there is previous evidence that they downplay the risk of cancer from alcohol consumption, in particular breast cancer. We analysed the cancer tweets from each source, to determine which specific cancers were mentioned across 2016 and 2017. It was only possible to include two of the industry-funded sources and two of the non-industry-funded sources as there were no relevant cancer-related tweets from other sources. We included only tweets from any source which mentioned cancer risk, and excluded tweets which did not specifically mention risk of cancer from alcohol consumption. The latter were typically tweets which included a Twitter handle from a cancer charity, but without specifically mentioning alcohol consumption. We were able to include tweets from industry-funded sources Drinkaware, and Drinkaware.ie, and from the independent charities Alcohol Concern and Alcohol Action Ireland; a total of 59 mentions of cancer from industry-funded sources, and 116 from independent alcohol charities (Supplementary Table S2).

The total number of tweets mentioning alcohol and cancer risk was lower for the two AI-funded bodies combined across both years (*n* = 59 (3.1%)) vs. the non-AI-funded bodies (*n* = 116 (9.48%)). When tweeting about cancer, the AI-funded bodies were significantly less likely to mention breast cancer (9/59 mentions of breast cancer: 15.25%) than non-AI-funded bodies (38/116; 32.76%) (Z = 2.47; 95%CI of difference = 3.62, 31.4; *p* (2-tailed) = 0.014).

Over half of Drinkaware’s cancer-related tweets in 2016 mentioned oral cancer. In four of these tweets, Drinkaware stated that the risk of oral cancer comes from ‘excessive’ drinking, which is inaccurate.

### 3.3. Content of Tweeted Images

We coded the visual content of the images included in tweets, comparing industry-and non-industry–funded sources. We examined how often women, children, and images of alcoholic beverages, as well as images of alcohol being drunk were represented Table 4. 

The industry-funded bodies were significantly more likely to show the relevant drinking, image or population group in every case. In each case most of this difference was due to Drinkaware tweets— (i.e., in each case Drinkaware was by far the largest group (see Supplementary Table S1)). For example where there was an image of people, 17.7% of Drinkaware tweets included only or mainly young women; the largest comparable percentage for a non-industry body was 2.7% (Alcohol Concern).

We found no evidence of these AI–funded bodies using images in their Twitter feed to extend drinking occasions (thus rejecting Hypothesis 6).

Table 5 summarises the study findings according to the eight main study hypotheses.

## 4. Discussion

Babor and Robaina (2013) in their analysis of the activities of alcohol industry CSR organisations, describe how “…there are common corporate interests across the spectrum of industry organizations, which sometimes conflict with public health and medical priorities but, at other times, are compatible with them” [41].

This analysis provides a clear analysis of the commonalities these organisations share with their corporate funders, and how they may conflict with public health priorities. It is clear firstly that these AI-funded bodies Twitter activities are consistent with the global alcohol producers’ commitments, as listed in Table 1 [42]. This activity prioritises behavioural aspects of drinking, with a particular focus on young women, rather than on specific health harms. This is consistent with previous evidence that the purpose of such bodies is the protection of the alcohol industry’s reputation and, in particular, to protect against the reputational harms caused by publicly visible alcohol-related antisocial behaviour (ASB), particularly in young women [18].

Consistent with this, these industry-funded organisations are significantly less likely to tweet about chronic health harms, cancers in particular. They are also much less likely to tweet (if at all) about issues which are likely to negatively impact on the alcohol industry, particularly alcohol advertising, sponsorship, and regulation of pricing (e.g., MUP). By selectively focusing on issues like ASB, AI-funded bodies’ Twitter activity may help shape perceptions as to what is needed to address alcohol-related harms; that is, education and individual responsibility, as opposed to more effective approaches, such as regulation of the industry and the conditions under which they supply and sell their products. This is a well-documented alcohol industry strategy, and it suggests that these organisations’ Twitter activities are closely aligned with that strategy [15,44].

The focus away from health harms, particularly cancer, by the alcohol industry-funded organisations is also consistent with what is already known. One other pattern also may be apparent. In the UK during 2016, (when most of the data were collected) there was considerable UK media discussion on breast cancer and alcohol, particularly in the early part of the year when the UK Chief Medical Officers (CMO’s) new alcohol consumption guidelines were released (on 6 January 2016). There was considerable media discussion of alcohol and breast cancer risk, following the discussion of this issue by the CMO. During this period the focus of tweets from the Drinkaware account were on oral cancer, rather than breast cancer, and most of their tweets about oral cancer tweets were confined to this period. Furthermore, the message conveyed was, at times, inaccurate, associating the risk with “excessive” drinking. The risk of oral cancer with alcohol consumption is in fact linear.

Industry-funded organisations’ pattern of tweeting about alcohol and pregnancy is also significantly different from non-industry-funded bodies (i.e., Hypothesis 3). It is known that AI-funded bodies misrepresent the evidence on the risks of alcohol consumption in relation to pregnancy and fertility [45], and provide inadequate and unclear information on pregnancy—for example, by smaller pregnancy warning labels on wine bottles (more likely to be drunk by women); and not using red (‘stop’) pregnancy warning labels [46]. The findings here are consistent with this: AI-funded bodies do not appear to use Twitter to raise awareness about pregnancy and fertility. It is possible that this is because it potentially has a negative effect on the female market, which is increasingly important to the alcohol industry [47,48].

In general this focus in industry messaging away from health harms and towards individual behaviours may also be seen as an attempt to (re)define or (re)capture the narrative, a recognised industry tactic across many industries [49]. In this case, it may reflect an alcohol industry framing of the issue of alcohol harms as one of individual responsibility, and ‘lack of control’ of one’s drinking, as opposed to a public health problem. This may explain the differences between the industry-funded and non-industry funded organisations, in which harms they discuss.

This framing may also be seen in the images included in the tweets. The AI-funded bodies were significantly more likely to use alcohol and drinking images. Alcohol/drinking images have been shown to act as a cue to alcohol craving and drinking [50,51,52], and it has also been found that alcohol information materials encouraging ‘responsible drinking’ can have the opposite effect, by encouraging drinking [7,31]. The use of images, in the absence of explicit advertising, and their impact is well documented by studies of tobacco industry (strategies. For example, that industry’s close relationship with Hollywood and the association between exposure to smoking imagery in films and smoking initiation and consumption are well established [53,54]. Working through front group Netnographica, the tobacco industry has also exploited social media platforms by paying young social media influencers to post images of smoking and cigarettes without disclosure of payment, thereby reaching millions of followers [55].

Overall, these suggest that there is a difference between the stated visions, values and missions of the AI-funded bodies, and their actual activities. For example, Drinkaware states that they will fulfil their vision of reducing alcohol-related harm by raising awareness of the harms of alcohol as well as providing impartial and evidence-based information [56]. These analyses show that their messages delivered via social media are very different from those of independent organisations, do not reflect many public health priorities, and appear to align with alcohol industry-interests.

Further, it is also notable that industry-funded organisations are more likely to tweet about Alcohol-free or low alcohol drinks, perhaps reflecting the rapid industry development of this market.

One implication of these differences is that industry-provided information may represent a risk to public health, if it selectively frames alcohol harms in particular ways, and misrepresents health information (as is known to be the case). Moreover lack of consistency across industry-funded and non-industry-funded sources of health information may cause doubt in the public mind, for example by appearing to show a lack of consensus on the health harms of alcohol consumption, or the levels of alcohol associated with specific harms.

The significant strength of this study is the direct, quantitative comparison with independent, non-industry-funded organisations, and the analysis of both text, and images. The main limitation is that we have no direct information on the intentions behind the Twitter activity—e.g., no internal documents or evidence of why certain words or images were chosen, nor how the use and content of Twitter accounts is managed and governed or how tweet content is developed within the organisations. We, therefore, make the assumption that the content of tweets reflect the aims and values of the organisations as opposed to individuals within them. We are also unable to confirm the prioritisation afforded to tweeting within each of the organisations’ communications approaches and whether this differs between organisations. However, our conclusions are supported by the fact that the findings are consistent with previous findings regarding AI activities and strategies.

There are also some potentially important areas for further research. Further detailed analysis of the imaging in AI tweets and websites is needed. We also noted instances where images and graphic design aspects of industry tweets appeared to subvert health information in the accompanying text. Further analysis of such ‘nudge’ type methods in industry social media activity and on their websites, and the impact these have on the user, would be very important.

## 5. Conclusions

This analysis provides further evidence that SAPROs such as Drinkaware and DrinkWise may be a mechanism by which alcohol industry framings are disseminated to the public. The concerns of these industry-funded bodies, as evidenced through their Twitter activity, closely mirror alcohol industry positions (e.g., the Global Alcohol Producers commitments) and strategies. These strategies are represented strongly in their tweets, particularly focusing on individual responsibility, rather than industry responsibility; not mentioning alcohol marketing, availability and pricing; under-representing health harms, and cancers in particular; focusing on young women because of the reputational risk to the industry caused by visible drunkenness in women; and a selective focus on the behavioural aspects of drinking. Claims by these bodies about their independence from industry are not supported by the evidence.

## Figures and Tables

**Table 1:** Global Alcohol Producers Commitments [13] and the areas for national action of the WHO Global Alcohol Strategy [14].

WHO Global Alcohol Strategy Areas for National Action	Global Alcohol Producers Commitments
Leadership, awareness and commitment	Reduce underage drinking
Health services response	Providing consumer information and responsible product innovation
Community action	Reducing drinking and driving
Drink driving policies and countermeasures	Working with retailers support to reduce harmful drinking
Availability of alcohol	Strengthening/expanding marketing codes of practice
Marketing of alcoholic beverages	
Pricing policies	
Reducing the negative consequences of drinking and alcohol intoxication	
Reducing the public health impact of illicit alcohol and informally produced alcohol	
Monitoring and surveillance	

**Table 2:** Twenty most common topics tweeted about in 2016 by alcohol industry-funded and non-industry-funded bodies (non-industry bodies shaded in grey) *n* (%).

Topic	Drinkaware	Drinkaware.ie	DrinkWise	AAI ^1^	Alcohol Concern	FARE ^2^	Total
Drinking too much	101 (12.1%)	14 (6.0%)	3 (3.4%)	17 (3.6%)	13 (1.8%)	40 (9.0%)	188 (6.7%)
Marketing, advertising, sponsorship or restrictions	0 (0.0%)	0 (0.0%)	0 (0.0%)	36 (7.6%)	42 (5.8%)	88 (19.8%)	166 (5.9%)
Drink driving	36 (4.3%)	41 (17.7%)	4 (4.5%)	64 (13.5%)	18 (2.5%)	1 (0.2%)	164 (5.8%)
Cancer	27 (3.2%)	11 (4.7%)	0 (0.0%)	34 (7.2%)	54 (7.4%)	2 (0.5%)	128 (4.6%)
Cutting down/cutting back	88 (10.6%)	16 (6.9%)	10 (11.2%)	7 (1.5%)	4 (0.5%)	0 (0.0%)	125 (4.5%)
Children/underage drinking	57 (6.8%)	7 (3.0%)	7 (7.9%)	9 (1.9%)	7 (1.0%)	27 (6.1%)	114 (4.1%)
Alcohol harms incl. dementia, diabetes, asthma, heart	23 (2.8%)	2 (0.9%)	0 (0.0%)	32 (6.7%)	29 (4.0%)	16 (3.6%)	102 (3.6%)
Calories/Obesity	89 (10.7%)	4 (1.7%)	0 (0.0%)	2 (0.4%)	3 (0.4%)	0 (0.0%)	98 (3.5%)
Teens/Parents	11 (1.3%)	65 (28.0%)	6 (6.7%)	5 (1.1%)	0 (0.0%)	0 (0.0%)	87 (3.1%)
Mental health	39 (4.7%)	3 (1.3%)	0 (0.0%)	22 (4.6%)	18 (2.5%)	0 (0.0%)	82 (2.9%)
Alcohol Pricing or Taxation or MUP ^3^	0 (0.0%)	0 (0.0%)	0 (0.0%)	37 (7.8%0	34 (4.7%)	9 (2.0%)	80 (2.9%)
Staying safe	55 (6.6%)	13 (5.6%)	8 (9.0%)	0 (0.0%)	0 (0.0%)	0 (0.0%)	76 (2.7%)
Pregnancy or fertility	10 (1.2%)	0 (0.0%)	1 (1.1%)	10 (2.1%)	9 (1.2%)	27 (6.1%)	57 (2.0%)
Alcohol guidelines	25 (3.0%)	12 (5.2%)	1 (1.1%0	4 (0.8%)	7 (1.0%)	0 (0.0%)	49 (1.7%)
Anger/Aggression	8 (1.0%)	0 (0.0%)	0 (0.0%)	6 (1.3%)	0 (0.0%)	26 (5.9%)	40 (1.4%)
Other peoples drinking	19 (2.3%)	0 (0.0%)	0 (0.0%)	18 (3.8%)	1 (0.1%)	1 (0.2%)	39 (1.4%)
Definition of units of alcohol	24 (2.9%)	11 (4.7%)	0 (0.0%)	0 (0.0%)	1 (0.1%)	0 (0.0%)	36 (1.3%)
Alcohol-free or low alcohol drinks	19 (2.3%)	1 (0.4%)	0 (0.0%)	0 (0.0%)	11 (1.5%)	0 (0.0%)	31 (1.1%)
Impact on emergency services	2 (0.2%)	5 (2.2%)	0 (0.0%)	5 (1.1%)	10 (1.4%)	9 (2.0%)	31 (1.1%)
Other	75 (9.0%)	23 (9.9%)	25 (28.1%)	51 (10.7%)	223 (30.5%)	89 (20.0%)	486 (17.3%)
**Total**	835						

^1^ Alcohol Action Ireland; ^2^ Foundation for Alcohol Research & Education; ^3^ Minimum Unit Pricing.

**Table 3:** Comparison of non-alcohol industry-funded charities and industry-funded charities by subject of tweet for twenty most common topics.

Topic	Industry-Funded	Non-Industry Funded	Chi-Squared	Z; Difference in % (95% CI ^1^)	*p* Value
Drinking too much	118 (10.2%)	70 (4.25%)	38.72	Z = 6.21; 5.96 (4.08–7.84)	<0.0001 ***
Marketing, advertising, sponsorship or restrictions	0 (0%)	166 (10.1%)	123.5	Z = 11.14; 10.1 (8.32–11.88)	<0.00001 ***
Drink Driving	81 (7.0%)	83 (5.0%)	4.83	Z = 2.2; 1.98 (0.22–3.74)	0.028 **
Cancers	38 (3.3%)	90 (5.5%)	7.33	Z = 2.74 2.2 (0.63–3.77)	0.007 **
Cutting down/cutting back	114 (9.9%)	11(0.7%)	135.1	Z = 11.58;9.2 (7.64–10.76)	<0.00001 ***
Children/underage drinking	71 (6.2%)	43 (2.6%)	21.81	Z = 4.67; 3.54 (2.05–5.03)	0.000003 ***
Alcohol harms incl. dementia, diabetes, asthma, heart disease	25 (2.2%)	77 (4.7%)	12.16	Z = 3.43; 2.47 (1.06–3.88)	0.0005 ***
Calories/Obesity	93 (8.1%)	5 (0.3%)	120.9	Z = 11.04; 7.8 (6.42–9.18)	<0.00001 ***
Teens/parents	82 (7.1%)	5 (0.3%)	104	Z = 10.22; 6.8 (5.5–8.1)	<0.00001 ***
Mental Health	42 (3.6%)	40 (2.4%)	3.5	Z = 1.81; 1.17 (−0.09–2.43)	0.061
Alcohol Pricing or Taxation or MUP	0 (0%)	80 (4.9%)	57.66	Z = 7.59; 4.85 (3.6–6.1)	<0.00001 ***
Staying safe while drinking	76 (6.6%)	0 (0%)	111.49	Z = 10.58; 6.6 (5.38–7.82)	<0.00001 ***
Pregnancy or fertility	11 (0.95%)	46 (2.8%)	11.5	Z = 3.41; 1.85 (0.79–2.91)	0.0007 ***
Alcohol guidelines	37 (3.2%)	11 (0.7%)	25.96	Z = 5; 2.5 (1.52–3.48)	0.0000004 ***
Anger/Aggression	8 (0.7%)	32 (1.9%)	7.52	Z = 2.75; 1.25 (0.36–2.14)	0.006 **
Other peoples drinking	19 (1.7%)	20 (1.2%)	0.93	Z = 0.98; 0.44 (−0.44–1.32)	0.34
What’s a unit	35 (3%)	1 (0.06%)	47.25	Z = 6.84; 2.94 (2.1–3.78)	<0.00001 ***
Alcohol-free or low alcohol drinks	20 (1.7%)	11 (0.7%)	7.04	Z = 2.64; 1.06 (0.27–1.85)	0.008 **
Impact of drinking on use of emergency services	7 (0.61%)	24 (1.46%)	4.48	Z = 2.11; 0.85 (0.06–1.64)	0.03 *
Other	123 (10.6%)	363 (22%)	61.22	Z = 7.85; 11.4 (8.56–14.24)	<0.00001 ***

* *p* < 0.05; ** *p* < 0.01; *** *p* < 0.001. ^1^ Confidence interval.

**Table 4:** Comparison of tweets by alcohol-industry-funded and non-alcohol-industry funded bodies, including different types of images.

Image Content	Alcohol Industry-Related Tweets *n* (%)	Non-Industry Tweets (*n* and % Showing that Image)	Chi-Squared (Uncorrected)	Z; Difference in % (95% CI)	*p*
Image of one or more women only	149/1155 (12.90%)	67/1649 (4.1%)	74.61	Z = 8.59; 8.8 (6.79–10.81)	<0.000001 ***
Children	71 (6.15%)	22 (1.33%)	49.07	Z = 7.02; 4.82 (3.47–6.17)	<0.000001 ***
Young adults	286 (24.76)	112 (6.79%)	180.09	Z = 13.42; 17.97 (15.35–20.59)	<0.000001 ***
Mainly young women	153 (13.25%)	31 (1.88%)	143.13	Z = 11.97; 11.37 (9.51–13.23)	<0.000001 ***
Alcoholic beverage	205 (17.75%)	58 (3.52%)	161.86	Z = 12.72; 14.23 (12.04–16.42)	<0.000001 ***
People drinking	121 (10.48%)	19 (1.15%)	124.49	Z = 11.16; 9.33 (7.69–10.97)	<0.000001 ***

*** *p* < 0.001.

**Table 5:** Summary of findings relating to the eight key hypotheses.

Hypotheses	Finding
H1: The topics covered by AI-funded bodies would be similar to the Global Alcohol Producers Commitments. (reducing under-age drinking; strengthening and expanding marketing codes of practice; providing consumer information and responsible product innovation; reducing drinking and driving; and enlisting the support of retailers to reduce harmful drinking).	AI–funded bodies were more likely to tweet about reducing underage drinking; alcohol-free and low-alcohol drinks (relevant to product innovation); and drinking and driving. Not enough tweets clearly attributable to marketing codes of practice, or enlisting the support of retailers to permit analysis. They were significantly more likely to mention information aspects of drinking—e.g., the alcohol guidelines, and knowing about units.
H2. That AI-funded tweets would have a focus on behavioural aspects of drinking and drink-related harms (i.e., visible antisocial behaviour, rather than chronic health harms).	AI-funded organisations were much more likely to tweet about behavioural aspects of drinking (e.g., drinking too much; ‘staying safe’; ‘cutting down/cutting back’).
H3. That AI-funded bodies would be less likely to tweet warning consumers about pregnancy and related issues, and about cancers.	AI-funded organisations were significantly less likely to tweet about pregnancy or fertility; cancers, and breast cancer specifically; and alcohol harms more generally.
H4. That AI-funded organisations’ Twitter communications would be primarily addressed to young women (because visible public drinking and alcohol-related anti-social behaviour in young women is a PR risk to the industry).	AI–funded organisations’ images were significantly more likely to include women, and young women specifically.
H5. That they would emphasis non-regulatory and self-regulatory initiatives.	AI-funded organisations were significantly less likely to mention taxation or pricing (e.g., minimum unit pricing); more likely to mention the alcohol guidelines, which are non-regulatory.
H6. That they would show evidence of normalisation of new drinking occasions (e.g., work drinks days), because of evidence that industry CSR ‘responsible drinking’ campaigns can have the dual effect of promoting drinking.	No clear evidence of this was observed in the data; the hypothesis was, therefore, rejected.
H7. Related to H6, that industry-funded organisations’ Twitter activity would be more likely to include alcohol, and drinking-related images.	Industry-funded organisations’ tweets were significantly more likely to show people drinking, and alcoholic beverages.
H8. For the analyses of images in the tweets, based on previous research and knowledge of AI priorities we hypothesised that industry-funded organisations’ images would be more likely to include women, and young women in particular; children; images of alcoholic drinks, and images of people drinking.	Industry-funded bodies (predominantly Drinkaware) were significantly more likely to show the relevant drinking, image or population group in every case.

## Supplementary Materials

The following are available online at https://www.mdpi.com/1660-4601/16/5/892/s1, Table S1. Full list of 45 topics coded, Table S2. Tweets mentioning cancer risk from alcohol during 2016 (*N* = number of tweets mentioning risk of cancer; *n* = number of mentions of specific cancer).
