# Supplementary material for: Alcohol Industry CSR Organisations: What Can Their Twitter Activity Tell Us about Their Independence and Their Priorities? A Comparative Analysis
Source: Int J Environ Res Public Health. 2019 Mar 12;16(5):892. doi: 10.3390/ijerph16050892 (PMC6427731; doi:10.3390/ijerph16050892)
Supplement: Supplementary file 1 [file ijerph-16-00892-s001.zip › Supplementary Tables.docx]

**Supplementary Table S1**

| **Table S1: List of 45 topics recoded (plus ‘Other’ category)** | | | Total |
| --- | --- | --- | --- |
|  |  |  |  |
| Topic recoded. NB "marketing restrictions" category includes advertising sponsorship and restrictions | -Alcohol labelling | Count | 15 |
|  |  | % within Source | 0.5% |
|  | -Marketing or advertising or sponsorship or restrictions | Count | 166 |
|  |  | % within Source | 5.9% |
|  | -Alcohol Pricing or Taxation or MUP | Count | 80 |
|  |  | % within Source | 2.9% |
|  | -Public Health Alcohol Bill (Alcohol Action Ireland) | Count | 44 |
|  |  | % within Source | 1.6% |
|  | Alcohol addiction | Count | 15 |
|  |  | % within Source | 0.5% |
|  | Pregnancy or fertility | Count | 57 |
|  |  | % within Source | 2.0% |
|  | Alcohol guidelines | Count | 49 |
|  |  | % within Source | 1.7% |
|  | Alcohol harms incl dementia, diabetes, asthma, CVD | Count | 102 |
|  |  | % within Source | 3.6% |
|  | Alcohol industry activities | Count | 30 |
|  |  | % within Source | 1.1% |
|  | Alcohol licensing | Count | 65 |
|  |  | % within Source | 2.3% |
|  | Alcohol poisoning | Count | 24 |
|  |  | % within Source | 0.9% |
|  | Alcohol related violence (incl DV) | Count | 21 |
|  |  | % within Source | 0.7% |
|  | Alcohol-free or low alcohol drinks | Count | 31 |
|  |  | % within Source | 1.1% |
|  | Anger/Aggression | Count | 40 |
|  |  | % within Source | 1.4% |
|  | Appearance | Count | 15 |
|  |  | % within Source | 0.5% |
|  | Availability/alcohol environment | Count | 2 |
|  |  | % within Source | 0.1% |
|  | Calories/Obesity | Count | 98 |
|  |  | % within Source | 3.5% |
|  | Cancer | Count | 128 |
|  |  | % within Source | 4.6% |
|  | Children of alcoholics | Count | 3 |
|  |  | % within Source | 0.1% |
|  | Children/underage drinking | Count | 114 |
|  |  | % within Source | 4.1% |
|  | Cutting down/cutting back | Count | 125 |
|  |  | % within Source | 4.5% |
|  | Drink Driving | Count | 164 |
|  |  | % within Source | 5.8% |
|  | Drinking too much | Count | 188 |
|  |  | % within Source | 6.7% |
|  | DrinkWise responsible drinking campaign | Count | 18 |
|  |  | % within Source | 0.6% |
|  | Dry humour | Count | 47 |
|  |  | % within Source | 1.7% |
|  | Dry January | Count | 163 |
|  |  | % within Source | 5.8% |
|  | Economic burden | Count | 6 |
|  |  | % within Source | 0.2% |
|  | Feeling good/not feeling good | Count | 12 |
|  |  | % within Source | 0.4% |
|  | Impact on emergency services | Count | 31 |
|  |  | % within Source | 1.1% |
|  | Liver disease | Count | 14 |
|  |  | % within Source | 0.5% |
|  | Mental Health | Count | 82 |
|  |  | % within Source | 2.9% |
|  | Mixing drink and drugs | Count | 6 |
|  |  | % within Source | 0.2% |
|  | Mixing with energy drinks | Count | 11 |
|  |  | % within Source | 0.4% |
|  | Moderate drinking/amount you drink | Count | 6 |
|  |  | % within Source | 0.2% |
|  | Offending | Count | 15 |
|  |  | % within Source | 0.5% |
|  | Other | Count | 486 |
|  |  | % within Source | 17.3% |
|  | Other peoples drinking | Count | 39 |
|  |  | % within Source | 1.4% |
|  | Positive trends | Count | 7 |
|  |  | % within Source | 0.2% |
|  | Regulation | Count | 13 |
|  |  | % within Source | 0.5% |
|  | Sexual harassment | Count | 24 |
|  |  | % within Source | 0.9% |
|  | Sleep/tiredness | Count | 22 |
|  |  | % within Source | 0.8% |
|  | Stay safe | Count | 76 |
|  |  | % within Source | 2.7% |
|  | Teens/parents | Count | 87 |
|  |  | % within Source | 3.1% |
|  | What's a unit | Count | 36 |
|  |  | % within Source | 1.3% |
|  | Women and alcohol | Count | 11 |
|  |  | % within Source | 0.4% |
|  | Young people drinking | Count | 16 |
|  |  | % within Source | 0.6% |
| Total | | Count | 2804 |
|  |  | % within Source | 100.0% |

**Supplementary Table S2: Tweets mentioning cancer risk from alcohol during 2016 (N=number of tweets mentioning risk of cancer; n=number of mentions of specific cancer)**

*Tweets during 2016:*

Drinkaware, 2016 (N=31): Mouth/throat/oral (n=17); Cancer in general, type unspecified (n=9); Liver (n=8); Breast (n=2); Bowel (n=1); Larynx (n=1).

Drinkaware.ie, 2016 (N=11): Cancer (unspecified) (n=4); Bowel (n=4); Breast (n=2); Stomach (n=2); Head and Neck (n=1).

Alcohol Concern, 2016 (N=54) Cancer (unspecified) (n=25); Breast (n=23); Bowel (n=13); Stomach (n=8); Mouth/Throat/Oral (n=16); Liver (n=9); Oesophagal, n=7; Pharynx (n=3); Larynx (n=4).

Alcohol Action Ireland, 2016 (N=13): Cancer (unspecified) (n=12); Breast (n=1).

*Tweets during 2017:*

Drinkaware, 2017 (N=9): Cancer (unspecified) (n=3); Breast, n=4; Bowel, n=1; Liver, n=1.

Drinkaware Ireland, 2017 (N=8): Cancer (unspecified) (n=5); Bowel/digestive, n=2; Breast, n=1; Liver (n=1); Pancreatic (n=1); Oesophagus (n=1); Gastric/Stomach (n=2).

Alcohol concern, 2017 (N=20): Breast, n=6; Cancer (unspecified) (n=13); Bowel/colorectal, n=1

Alcohol Action Ireland, 2017 (N=29): Cancer (unspecified) (n=16); Breast, n=8; liver, n=8; Gastric/stomach, n=3; Pancreatic, n=2; Mouth/Throat, n=11; Bowel (n=4); Oesophagus (n=4); Larynx (n=4).
